# Hydrogel Scaffolds to Deliver Cell Therapies for Wound Healing

**DOI:** 10.3389/fbioe.2021.660145

**Published:** 2021-05-03

**Authors:** Dharshan Sivaraj, Kellen Chen, Arhana Chattopadhyay, Dominic Henn, Wanling Wu, Chikage Noishiki, Noah J. Magbual, Smiti Mittal, Alana M. Mermin-Bunnell, Clark A. Bonham, Artem A. Trotsyuk, Janos A. Barrera, Jagannath Padmanabhan, Michael Januszyk, Geoffrey C. Gurtner

**Affiliations:** Division of Plastic and Reconstructive Surgery, Department of Surgery, Stanford University School of Medicine, Stanford, CA, United States

**Keywords:** hydrogel, cell therapy, wound healing, fibrosis, stem cell

## Abstract

Cutaneous wounds are a growing global health burden as a result of an aging population coupled with increasing incidence of diabetes, obesity, and cancer. Cell-based approaches have been used to treat wounds due to their secretory, immunomodulatory, and regenerative effects, and recent studies have highlighted that delivery of stem cells may provide the most benefits. Delivering these cells to wounds with direct injection has been associated with low viability, transient retention, and overall poor efficacy. The use of bioactive scaffolds provides a promising method to improve cell therapy delivery. Specifically, hydrogels provide a physiologic microenvironment for transplanted cells, including mechanical support and protection from native immune cells, and cell–hydrogel interactions may be tailored based on specific tissue properties. In this review, we describe the current and future directions of various cell therapies and usage of hydrogels to deliver these cells for wound healing applications.

## Introduction

Skin acts as a critical protective barrier against external agents (Saghazadeh et al., 2018). After cutaneous injury, such as from a burn or cut, the normal skin structure is disrupted, and the resultant wound progresses through a coordinated cascade (“wound healing”) of molecular and cellular processes to restore or replace the damaged tissue (Reinke and Sorg, 2012; Gonzalez et al., 2016).

To improve outcomes after cutaneous injury, researchers have investigated the use of cellular therapies to treat wounds, utilizing a wide array of cell types such as fibroblasts, endothelial cells, platelets, myeloid cells, and stem cells. Several products utilizing adult cells are also already commercially available including Dermagraft^®^ and Apligraf^®^ (Gentzkow et al., 1996; Edmonds, 2009). Multipotent stem cells have become an increasingly attractive choice for cell-based therapy due to their proliferative potential, differentiation capacity, and ability to secrete trophic factors and extracellular matrix (ECM) components important for wound healing (You and Han, 2014). The primary limitation with delivering cell therapies into cutaneous wounds has been low viability and transient engraftment of the transplanted cells. In order to maximize cell viability, a supportive microenvironment must be established to improve survival of these transplanted cells (Kamoun et al., 2017).

Biological scaffolds, such as hydrogels, provide an ideal, physiochemical mimetic of native ECM that can be utilized as a delivery vehicle for cells (Geckil et al., 2010). Hydrogels are hydrophilic gels with a three-dimensional structure that rapidly swell in water to form a semi-solid. The water content of hydrogel matrices exceeds 90%, ideal for hydrating and maintaining a supportive environment within the wound bed that accelerates angiogenesis, increases breakdown of dead tissue, prevents cell and tissue death, and even alleviates pain (Field and Kerstein, 1994). The biophysical and biochemical properties of the hydrogel can be tuned to adjust the microenvironment to support a variety of cell types (Farhat et al., 2019).

In this review, we detail the use of hydrogels to deliver stem cells for wound healing. We first discuss the advantages and disadvantages of delivering various cell types to improve wound healing. We then discuss the use of bioactive scaffolds and hydrogels to deliver these cells, including technical considerations, such as material selection for hydrogel synthesis, maintaining cell viability during hydrogel gelation, tailoring hydrogel–cell interactions, and preventing cell entrapment by modifying porosity and degradation. Finally, we provide our view of the most promising cell and hydrogel candidates for wound healing and discuss future strategies to accelerate wound healing and improve the quality of tissue repair.

## Overview of Wound Healing

**FIGURE 1:**
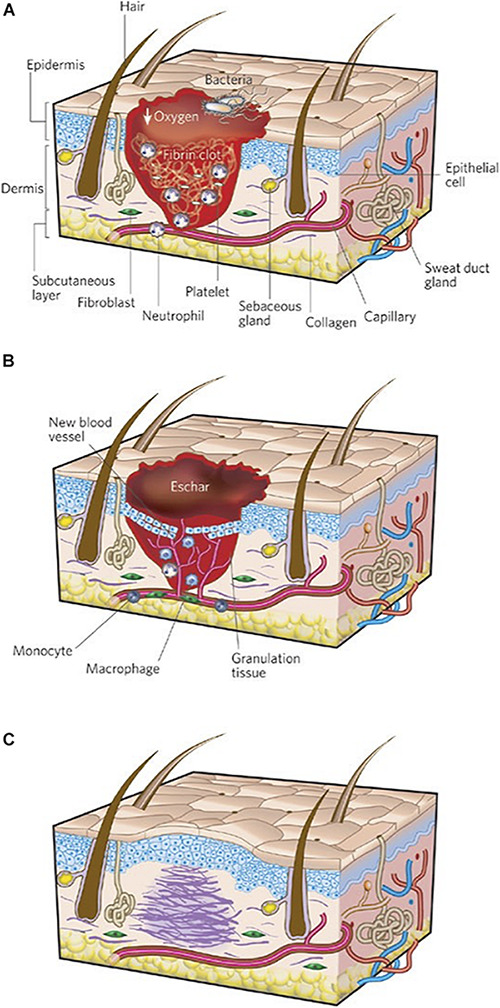
Three stages of wound repair. The three phases of wound repair consist of **(A)** inflammation, **(B)** new tissue formation, and **(C)** remodeling. **(A)** The inflammatory phase lasts until about 48 h after injury. Depicted is a skin wound at about 24–48 h after injury. The wound is characterized by a hypoxic (ischemic) environment in which a fibrin clot has formed. Bacteria, neutrophils, and platelets are abundant in the wound. Normal skin appendages (such as hair follicles and sweat duct glands) are still present in the skin outside the wound. **(B)** New tissue formation occurs about 2–10 days after injury. Depicted is a skin wound at about 5–10 days after injury. The majority of cells from the previous stage of repair have migrated from the wound, and new blood vessels now populate the area. An eschar (scab) has formed on the surface of the wound, and the migration of epithelial cells can be observed under the eschar. **(C)** Remodeling lasts for a year or longer. Depicted is a skin wound about 1–12 months after repair. Disorganized collagen has been laid down by fibroblasts that have migrated into the wound and contracted the wound. The re-epithelialized wound is slightly higher than the surrounding surface, and the healed region does not contain normal skin appendages. Figure adapted with permission from Figure 1 of Gurtner et al. (2008).

Wound healing proceeds through three sequential phases of inflammation, new tissue formation, and remodeling (Figure 1). Immediately after injury, blood and lymphatic fluid enters the wound site, activating coagulation pathways to facilitate platelet aggregation and achieve hemostasis (Gonzalez et al., 2016). During inflammation, inflammatory cells such as neutrophils and monocytes are recruited from the circulation to decontaminate the wound site through phagocytosis of cellular debris and bacteria (Guo and Dipietro, 2010; Schultz et al., 2011). Monocytes may differentiate into macrophages, a heterogenous and highly plastic cell population that may further differentiate into pro- or anti-inflammatory subtypes and are believed to be critical mediators of the cellular response during all stages of soft tissue injury (Gurtner et al., 2008). During new tissue formation, keratinocytes migrate over the injury to initiate re-epithelialization, fibroblasts and myofibroblasts deposit collagen and granulation tissue, and myofibroblasts contract and facilitate bringing the wound edges closer together (Schultz et al., 2011). In the remodeling phase (final and longest stage), wound contraction peaks and collagen is continually synthesized and reorganized (Gonzalez et al., 2016). These phases occur in a carefully coordinated fashion, and aberrancies in this tightly regulated process result in delayed or impaired wound healing (Gurtner et al., 2008).

## Clinical Significance of Wound Healing

**FIGURE 2:**
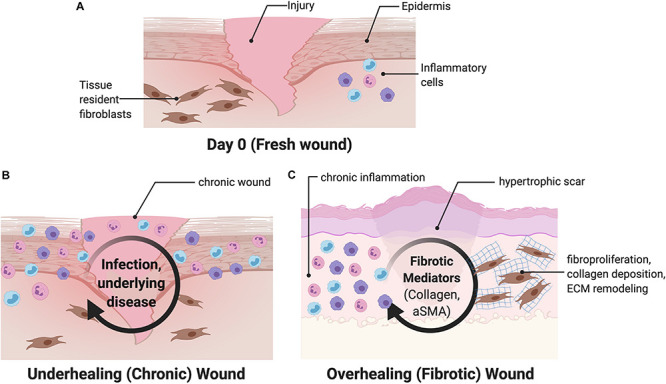
Overview of wound healing. **(A)** After an initial wound, both circulating and tissue resident cells are recruited to the wound, including fibroblasts and inflammatory cells. **(B)** Chronic wounds are characterized by interruptions and subsequent prolongation of the wound healing process. **(C)** Fibrotic wounds are characterized by upregulation of myofibroblast differentiation and increased collagen production and may be driven by mechanical signaling. aSMA, alpha smooth muscle actin.

Abnormalities in wound healing can either lead to “over healing,” resulting in excessive fibrosis, or “under healing,” leading to a chronic, non-healing wound (Frykberg and Banks, 2015). Infection, chronic inflammation, or vascular dysfunction delay wound closure and generate chronic wounds including arterial and venous ulcers, diabetic wounds, and pressure-related ulcers (Figures 2A,B) (Demidova-Rice et al., 2012; Frykberg and Banks, 2015; Xiang et al., 2019). Chronic wounds constitute a source of significant morbidity for the aging global population and a sizeable economic burden to the healthcare system (Jarbrink et al., 2017), with tens of billions of dollars spent annually on wound treatment (Nussbaum et al., 2018). Treatment of chronic wounds involves debridement of all necrotic tissue and selection of appropriate wound dressings that take into account the level of moisture, amount of wound exudate, presence of infection, and quality of the wound bed (Dabiri et al., 2016). In general, traditional topical therapies are geared toward facilitating a clean, moist environment conducive to healing.

In contrast, large surface area injuries such as burns almost always result in excessive fibrosis and hypertrophic scar (HTS) formation, which compromise normal function and result in severe morbidity to those affected (Berman et al., 2008; Gurtner et al., 2008). These scars are characterized by upregulation of myofibroblast differentiation, characterized by increased alpha smooth muscle actin (aSMA) signaling, and increased collagen production, driven by increased mechanotransduction (Figure 2C) (Wong et al., 2011a; Ma et al., 2018). Human fetuses display the ability to regenerate skin wounds without scar formation, unlike in adults, where scars develop from most skin wounds through the partial contributions of fibrotic and regenerative processes. The underlying mechanisms determining the fibrotic or regenerative fates of healing wounds remain unclear.

Management of patients with severe wounds is a long-term process that must address the local wound as well as the systemic, psychologic, and social consequences of the injury (Atiyeh et al., 2007; Chua et al., 2016; Zhu et al., 2016). Currently there are no standardized therapeutic treatment options for patients to address either excessive fibrosis or chronic wound formation. The use of some therapeutic agents, such as cytokine-based approaches, lacks strong scientific evidence, and many are based on case studies with mixed success (Meier and Nanney, 2006; Block et al., 2015; Rose and Chan, 2016). Newer cell-based therapies that target the underlying cellular and molecular processes of wound healing may provide a promising improvement for wound care.

## Cell Based Therapies

Cellular therapy refers to the transplantation of cells to replace or repair damaged tissue. Although therapeutics may come in the form of small molecules, biologics (e.g., antibodies, growth factors, cytokines, hormones), or cells, only cells may sense the cues in the wound healing environment and respond in complex fashions (Fischbach et al., 2013). Cells respond to the environmental cues, make decisions (proliferation, increased secretion, etc.), and then provide precise, dynamic control over extended time periods (Fischbach et al., 2013). When utilizing cell therapies, researchers must be sure that the complex benefits from delivering cells are required, instead of simpler small molecules and biologics that provide controlled, static treatments.

These cells may be autologous or allogeneic and can even be engineered to express unique ligands and target-specific receptors (Zakrzewski et al., 2019; Srifa et al., 2020). While autologous cells are well-tolerated at the injury site, harvesting enough cells can be challenging, and both time and money are required to expand and culture a sufficient quantity of cells needed for the therapy. Allogeneic cells are easier to obtain and manufacture, but the immune response is a potential impediment (Chidgey et al., 2008). Interestingly, some recent studies have found that transplantation of xenogeneic cells may attenuate fibrosis; however, this has not yet been fully explored for human therapy (Cargnoni et al., 2009). In the context of wound healing, many different cell types have been transplanted into wound beds including keratinocytes, fibroblasts, platelets, bone marrow derived mesenchymal stromal cells, and adipose derived stromal cells (You and Han, 2014). Broadly, stem cells appear to be the most promising cell-based therapy currently under investigation due to their low immunogenicity, secretion of trophic factors, and ability to differentiate into a wide range of cell types. Here, we discuss these cells in the context of cell therapies (Table 1).

**TABLE 1:** Cell therapies tested for wound healing.

**Cell type**	**Benefits**	**Limitations**	**Citations**
Keratinocytes	– May be harvested from a skin biopsy and expanded in culture to form a large sheet of epidermis that improves wound closure and epithelialization	– Unable to produce a robust extracellular matrix – Efficacy reduced in chronic wounds compared to acute wounds	Gentzkow et al., 1996; You and Han, 2014; Thamm et al., 2015; da Silva et al., 2019
		– Efficacy reduced in chronic wounds compared to acute wounds	
Fibroblasts	– Directly deposit extracellular matrix proteins	– Allogeneic fibroblast treatments have shown reduction in cell cryopreservation viability by almost 50% and inhibits protein production by 70–98%	Edmonds, 2009; You and Han, 2014
	– Shown to treat facial defects following skin cancer resection with minimal scar formation		
Macrophages and monocytes	– Improves rate of murine wound healing in both wild-type and diabetic mice, with no adverse effect on the quality of repair. – Increased angiogenesis in mice	– Did not improve the quality of the healed tissue in terms of tensile strength, scar formation, or collagen density	Hu et al., 2017
ASCs	– Easily obtained in large quantities	– No consensus on a common isolation protocol that is clinically feasible, and which would ensure reproducible results	Strioga et al., 2012; Hassan et al., 2014; Koenen et al., 2015; Bertozzi et al., 2017; Moon et al., 2019
	– Shown to improve wound healing by promoting angiogenesis, secreting paracrine signaling molecules and extracellular matrices, and differentiating along multiple cell lineages		
	– Multifaceted ability to respond to the changing wound healing phases		
Bone marrow MSCs (BM-MSCs)	– Capacity to differentiate into multiple cell types	– High donor site morbidity and low yield	Chen et al., 2009; Kim and Suh, 2010; Lian et al., 2014; You and Han, 2014; Isakson et al., 2015; Li et al., 2015; Tartarini and Mele, 2015; Liu et al., 2017; Pittenger et al., 2019
	– Reduce inflammation by diminishing cytokine expression and inflammatory cell chemotaxis	– Require *ex vivo* expansion prior to their application	
	– Promote neovascularization and recruit endogenous stem cells to the wound site	– Harvesting BM-MSCs is painful with donor site morbidity	
Umbilical cord-derived MSCs (UC-MSCs)	– Easily derived from the umbilical cord	– Requires significant *ex vivo* expansion over time	Nagamura-Inoue and He, 2014
	– Great rate of self-renewal	– Suffer from extensive phenotypic drift	
	– Primitive stem cells, with greater proliferative and immunosuppressive capabilities compared to other MSCs		

### Non-stem Cell-Based Therapies

Many past studies have investigated the ability of fully differentiated adult cells to improve wound healing. Keratinocytes, both allogeneic and autologous, have been used to cover wounds for over three decades (You and Han, 2014). They can be harvested from a skin biopsy and expanded in culture to form a large sheet of epidermis that improves wound closure and epithelialization (You and Han, 2014; da Silva et al., 2019). However, keratinocytes are themselves unable to produce a robust extracellular matrix (You and Han, 2014), and their efficacy appears to be significantly reduced in chronic wounds compared to acute wounds. Thamm et al. (2015) found that acute wound exudate supported keratinocyte proliferation over longer time intervals, while chronic wound exudate continually suppressed keratinocyte proliferation and migration.

Fibroblasts are mesenchymal cells crucial to the healing process and have frequently been explored as a potential cell-based therapy. Unlike keratinocytes, fibroblasts directly deposit extracellular matrix proteins such as collagens or proteoglycans. In a study using autologous fibroblasts seeded on a hyaluronic acid sheet to treat facial defects following skin cancer resection, all recipients healed with minimal scar formation (You and Han, 2014). By contrast, allogeneic fibroblast treatments have shown less promise, as any cell cryopreservation reduces viability by almost 50% and inhibits protein production by 70–98% (You and Han, 2014). Macrophages and monocytes have also been found to improve murine wound healing, although they did not improve the quality of the healed tissue in terms of tensile strength, scar formation, or collagen density in this study (Hu et al., 2017).

Apligraf^®^ and Dermigraft^®^ are tissue engineered products used in the clinic that contain living human cells (keratinocytes and fibroblasts, respectively) seeded within an ECM matrix (Gentzkow et al., 1996; Edmonds, 2009). Clinical trial studies demonstrate that use of these seeded scaffolds improved healing outcomes, such as wound closure, compared to control wounds. However, none of these studies compared the effects of the seeded scaffolds to unseeded scaffolds, and additional studies have found that the seeded cells are rejected and degraded within 1–2 weeks (Griffiths et al., 2004). While the use of each of these fully differentiated cell types provides some benefits, no one cell type provides all the necessary functions needed during wound healing, including producing ECM, promoting angiogenesis, and re-epithelialization. Furthermore, these allogeneic cell types may produce immune sensitization (Trainor et al., 2014).

### Benefits of Stem Cell Therapies Compared to Fully Differentiated Cells

Stem cells maximize the benefits of cell therapies by having reduced immunogenicity and increased therapeutic benefit, including stimulation of re-epithelialization and wound closure, ECM production, and angiogenesis (da Silva et al., 2019). MSCs can differentiate into many cell types and secrete trophic factors that promote healing (Dabiri et al., 2013; Hu et al., 2018). MSCs can be readily isolated from multiple tissue types and have been shown to accelerate and enhance wound healing by secreting beneficial cytokines, recruiting macrophages, inducing angiogenesis, and restoring sebaceous glands and hair follicles (Isakson et al., 2015; Kosaric et al., 2019). MSCs have immunosuppressive properties by releasing PGE2, galectin-1, HLA-G5, and indoleamine 2,3-dioxygenase (IDO) (Nagamura-Inoue and He, 2014), and low immunogenic characteristics, characterized by a lack of HLA-DR and low expression of MHC Class I molecules (Nagamura-Inoue and He, 2014). Additionally, even after up to 20–30 rounds of division, MSCs retain stem-like properties (You and Han, 2014; Li et al., 2015).

**FIGURE 3:**
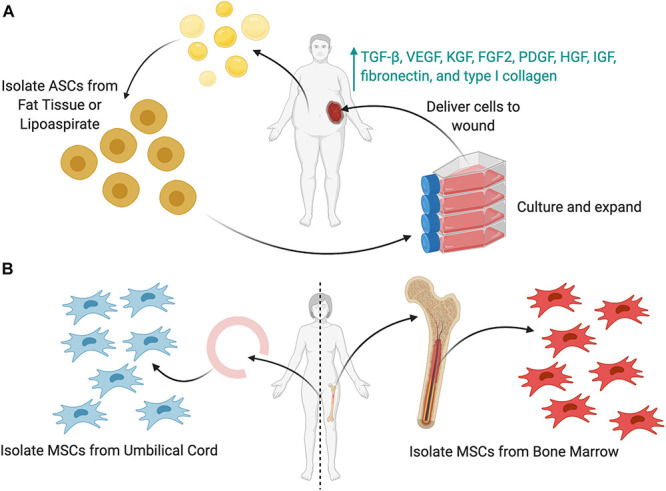
Sources of stem cells to be used as cellular therapies. **(A)** Adipose-derived stem cells (ASCs) can be harvested from either lipoaspirate or fat tissue. These cells (or MSCs) can then be cultured *in vitro*, expanded in number, and then delivered to the wound as a cell therapy. To enhance the cell delivery, several delivery techniques (shown later) may be used. **(B)** Mesenchymal stromal cells (MSCs) can be harvested from either the umbilical cord or the bone marrow.

Adipose-derived stromal cells (ASCs) are a subtype of MSCs studied extensively for wound healing. ASCs are easily obtained in large quantities via liposuction and surgical excision of fat tissue, with any given amount of adipose tissue yielding up to 40 times more stem cells than the same amount of bone marrow (Hassan et al., 2014). ASCs have been shown to improve wound healing by promoting angiogenesis, secreting paracrine signaling molecules and extracellular matrices, and differentiating along multiple cell lineages (Figure 3A) (Tartarini and Mele, 2015). ASCs secrete factors such as TGF-β, VEGF, KGF, FGF2, PDGF, HGF, IGF, fibronectin, and type I collagen, which enhance epithelial migration and dermal fibroblast proliferation (Kim et al., 2009; Hassan et al., 2014; Tartarini and Mele, 2015). ASCs cultured in acute wound fluid demonstrated increased proliferation and migration, necessary to increase cell numbers and facilitate wound closure during the early phases of healing. Meanwhile, ASCs cultured in chronic wound fluid showed increased expression of VEGF, bFGF, and MMP9, factors necessary to promote angiogenesis and mediate fibroblast growth (Koenen et al., 2015). These findings demonstrate the multifaceted ability of ASCs to respond to the changing wound healing phases.

Bone marrow MSCs (BM-MSCs) are found in the bone marrow stroma and are capable of differentiating into osteoblasts, adipocytes, myoblasts, and neurons (Figure 3B) (Lian et al., 2014; You and Han, 2014). BM-MSCs can also differentiate into multiple types of skin cells, such as keratinocytes, endothelial, pericytes, and monocytes, to release cytokines and hematopoietic factors including VEGF, angiopoietin-1, IGF-1, EGF, KGF, and stromal derived factor-1 (SDF-1) (Kim and Suh, 2010; Liu et al., 2017). BM-MSCs also reduce inflammation by reducing cytokine expression and inflammatory cell chemotaxis (Li et al., 2015). Chen et al., treated wounds with BM-MSCs and observed a decrease in CD45^+^ leukocytes, CD3^+^ lymphocytes, and CD8^+^ T-cells in an excisional wound mouse model (Chen et al., 2009; Li et al., 2015). Moreover, BM-MSCs promote neovascularization and recruit endogenous stem cells to the wound site, thereby accelerating the healing process (Lian et al., 2014).

Harvest of BM-MSCs is typically derived from bone marrow aspirate from the iliac crest, which is painful, associated with donor site morbidity, and often results in insufficient cell yield (Isakson et al., 2015; Tartarini and Mele, 2015). Early *in vitro* and animal studies of BM-MSCS quickly transitioned into human clinical trials for a multitude of clinical applications (Pittenger et al., 2019). Unfortunately, the clinical application of BM-MSCs has been limited by two factors. First, harvesting BM-MSCs is a painful process for patients to undergo and carries the risk of donor site morbidity (Isakson et al., 2015). Second, BM-MSCs require *ex vivo* expansion prior to their application due to the relatively low yields acquired after isolation. During cell expansion, MSCs are cultured on flat, unphysiological, and stiff materials such as tissue culture plastic or glass. These stiff substrates promote upregulation of a series of mechanotransduction genes, with increasing culture time leading to increased differentiation into osteogenic phenotypes (Yang et al., 2014). However, these substrates do not recapitulate the native cellular environment, in which cells are receiving signals in all three dimensions from native ECM (Caliari and Burdick, 2016). Yang et al. (2014) found that hMSCs possess a “mechanical memory,” in which hMSCs cultured for a longer time on plastic demonstrated higher mechanotransduction activation (through the YAP pathway) and osteogenic phenotypes, even after they had been seeded into more physiologic, soft hydrogels. Increased time in culture and increased passages will also influence cell phenotype, shape, morphology, and transcription (Yao et al., 2006), and the exact stiffness of the culture substrate will also affect factors such as secretion of growth factors and proliferation (Ogle et al., 2020). For wound healing, we have found that about 250,000 cells should be delivered to a 0.5 cm^2^ wound (Barrera et al., 2021; Srifa et al., 2020), and others have found that about 1–3 million cells per kg are needed for systemic injection of MSCs in human clinical trials (Capelli et al., 2015). Overall, these would require at least two cell passages to properly expand cell levels for therapy.

Umbilical cord-derived MSCs (UC-MSCs) can be harvested from cord blood and umbilical vein subendothelium, as well as the perivascular zone, intravascular zone, and sub-amnion of the Wharton jelly (Figure 3B) (Nagamura-Inoue and He, 2014). These are easily derived from the umbilical cord, which is generally discarded after birth and readily available (Nagamura-Inoue and He, 2014). UC-MSCs have a gene expression profile similar to that of embryonic stem cells and possess a greater rate of self-renewal compared to BM-MSCs, allowing for ease of harvest and expansion. UC-MSCs express markers such as CD13, CD29, CD73, CD90, CD105, and HLA-ABC and produce three times more collagen than BM-MSCs (Nagamura-Inoue and He, 2014). Additionally, they are more primitive stem cells, with greater proliferative and immunosuppressive capabilities compared to other MSCs. Although UC-MSCs are easier to collect and maintain viability *in vitro* longer than BM-MSCs, BM-MSCs may be initially isolated in higher quantities (up to fivefold more from explanted BM compared to an explanted umbilical cord) (Capelli et al., 2015). Thus, UC-MSCs require significantly more *ex vivo* expansion over time, leading to more phenotypic drift that reduces their therapeutic efficacy over time faster (Nagamura-Inoue and He, 2014; Isakson et al., 2015).

From a practical standpoint, ASCs are the most easily accessible and can be isolated in large quantities with minimal patient morbidity (Bertozzi et al., 2017). To date, only a few clinical trials have studied ASCs in the context of wound healing; however, the preliminary results appear promising (Moon et al., 2019). Both ASCs and BM-MSCs have important similarities, such as multi-lineage potential, morphology, telomerase activity, gene expression, and similar cell surface markers such as CD10, CD13, CD29, CD44, CD54, CD71, CD90, CD105, CD106, CD117, and STRO-1 (Hassan et al., 2014; Isakson et al., 2015). Although there exist some differences between ASCs and BM-MSCs, their common secretion profiles, angiogenic potential, gene expression profiles, and clinical data support the use of both BM-MSCs and ASCs in wound healing (Strioga et al., 2012). Certain wound pathologies may favor the use of one cell population over the other, so additional research will be required to further clarify the specific advantages and disadvantages for each situation.

### Potential for Engineered-Cell Therapies

New cell-based therapy approaches that utilize genome editing have also opened new and exciting avenues to treat previously intractable diseases. Transducing hematopoietic stem cells of β-thalassemia patients with a functional β-globin locus has been shown to eliminate the need for long-term red-cell infusions in a subset of patients with severe β-thalassemia (Thompson et al., 2018). Moreover, genetically modifying autologous patient-derived T cells with a chimeric antigen receptor (CAR) leads to impressive response rates in patients with hematologic malignancies (Raje et al., 2019). Precise genome editing via new CRISPR-based technologies may allow for targeted knock-out or knock-in of genes that are key regulators of signaling pathways involved in wound healing (Adli, 2018). For example, gene editing of MSCs could increase their secretion of growth factors such as PDGF and VEGF that are beneficial to wound healing by increasing angiogenesis (Srifa et al., 2020). As the field of gene editing grows, these new techniques could be used to further boost the potential of stem cell delivery, making these already beneficial cell types even more efficient. These early studies point to the promising future potential for genetic engineering platforms to develop novel cellular therapies that both accelerate as well as improve the quality of wound healing.

## Cell Delivery Methods

Although many cells show promising capabilities in wound healing, these beneficial effects can be limited by the efficacy of the delivery. Injection-based delivery of stem cells suspended in solution results in rapid cell death, low viability, and transient engraftment (Rustad and Gurtner, 2012; Rustad et al., 2012; Tartarini and Mele, 2015; Ma et al., 2018). Wu et al. (2007) injected BM-MSCs into an excisional wound model and found that engraftment dropped from 28% at 7 days to 7.6% at 14 days and then 2.5% at 28 days. Low viability and transient engraftment may be due to factors such as high shear stresses during injection, lack of extracellular matrix to bind and interact with upon injection, leakage from the site, mechanical washout of cells, or exposure of cells to inflammation and reactive oxygen species (ROS) present within the wound (Li and Mooney, 2016). Optimization of the cell delivery method can maximize cell-tissue interactions to both elicit the most effective responses and promote viability of stem cells in a hostile wound microenvironment (Falanga et al., 2007) (Table 2).

**TABLE 2:** Different scaffolds to deliver cell therapies for wound healing.

**Delivery method**	**Benefits**	**Limitations**	**Citations**
Alginate hydrogel	– Mechanical properties of hydrogel can be tuned	– Limited long-term stability in physiologic conditions	Percival and McCarty, 2015; Aderibigbe and Buyana, 2018; Salehi et al., 2020; Zhang and Zhao, 2020; Zhang et al., 2020
	– Establish a robust microenvironment for cells	– Must be modified with an adhesive ligand	
Collagen hydrogel	– Primary organic constituent of native ECM	– Damage to its covalent cross-links upon extraction weakens hydrogels, which can then disintegrate on handling or under the pressure of surrounding tissues *in vivo*.	Helary et al., 2012; Chattopadhyay and Raines, 2014; Chen et al., 2018; Stoica et al., 2020
	– Highly biocompatible and cytocompatible, amenable to cell adhesion without modification		
Fibrin hydrogel	– Natural role as a matrix involved in hemostasis and wound healing	– Fibrin can be especially susceptible to protease-mediated degradation	Ahmed et al., 2007; Janmey et al., 2009; Moreno-Arotzena et al., 2015; Murphy et al., 2017
	– Can trigger encapsulated cells to secrete ECM components and reparative growth factors		
Hyaluronic acid (HA) hydrogel	– Chemical tunability	– In modifications like cross-linked HA-aldehyde or HA-amine derivatives, there are disadvantages: the modification procedure involves many synthesis and purification steps, and the crosslinking chemistries that occur upon mixing are hard to control and yield inconsistent gels	Baier Leach et al., 2003; Silva et al., 2016
	– Favorable mechanical properties, biocompatibility, and biodegradation capacity		
Poly(dimethylsiloxane) (PDMS)	– Fosters viability and proliferation of seeded ASCs	– Poor biocompatibility	Schaffer et al., 1994; Razavi and Thakor, 2018
Poly-(ethylene glycol) (PEG)	– Versatility in chemical modification and ability to finely tune mechanical properties	– Synthesized in combination with natural polymers or biomimetic peptides as lack the biochemical properties for cellular interaction	Zhu and Marchant, 2011
Poly(lactic-co-glycolic acid) (PLGA)	– Extensively studied	– Poor biocompatibility	Uematsu et al., 2005; Sadeghi-Avalshahr et al., 2017
	– One of the most widely used polymers for materials science engineering applications	– Challenging to fixate within wound bed	
Poly(methyl methacrylate) (PMMA)	– Highly crosslinked gels possess longer degradation times	– In general, highly crosslinked gels possess longer degradation times	Henderson et al., 2010
Pullulan-collagen hydrogel	– Best approximate the porous ultrastructure of native reticular ECM	– It is possible that the hydrogel microenvironment is hypoxic	Wong et al., 2011b; Rustad et al., 2012
	– Easy engineering of the mechanical properties		
	– Able to support the growth of multiple cell types		
	– Minimal rejection and favorable biomaterial-tissue integration		
Gelatin hydrogel	– Excellent biocompatibility	– Accelerated biodegradation compared to other hydrogels	Kang and Park, 2021
	– Ease of chemical modification	– Variation between synthesized bathes	
		– Weak mechanical properties	

### Biomaterial Scaffolds for Cell Delivery in Wound Healing

Biologic scaffolds may serve as a delivery vehicle that remains viable, stable, and uncompromised within the harsh environment of the wound bed to maximize the potential of delivered cells. These ‘biomaterials’ have ECM structures similar to physiologic tissue where cells can be seeded to improve viability and retention. Biomaterials may broadly be defined as any ‘*material intended to interface with biological systems to evaluate*, *treat*, *augment, or replace any tissue*, *organ or function of the body’* (Williams, 2009). These biomaterials may be procured with techniques such as decellularization or immunomodulation of existing tissue (Ott et al., 2008); electrospinning, which mimics extracellular matrix structure; or triphasic culture, which mimics physiologic orthopedic interfaces (Spalazzi et al., 2006). These materials may be further modified through microtopography to provide signal cues for cellular differentiation (Downing et al., 2013).

“Decellularizing” native organs involves using a series of detergents and washes to remove all cellular material from the organ but retain most of the remaining extracellular matrix (Ott et al., 2008). The matrix can be re-seeded with a patient’s own cells to mitigate rejection of the resultant implanted organ. Ott et al. (2008) demonstrate proper contractile and pump function of decellularized murine and rat hearts re-seeded with new cells. Sullivan et al. (2012) similarly re-seeded decellularized kidneys with renal cells. For cell therapies, some have decellularized tissue such as porcine small intestine submucosa to re-seed them with cells such as tenocytes to treat rotator cuff defects (Chen et al., 2007). Milan et al. (2016) decellularized human skin samples and re-seeded them with human umbilical cord perivascular cells (HUCPVCs) to find that these cell-seeded scaffolds could improve wound closure and upregulate angiogenesis in a murine model. The HUCPVCs served as an alternative source of MSCs with higher proliferative rate and better cell yield. Cumulatively, these findings demonstrate that decellularized matrices could be successfully used as a vehicle to deliver cells to wounds.

One of the most commonly studied biomaterials is poly(dimethylsiloxane) (PDMS), a silicone material that is bio-inert and easy to produce. Various studies have found that cells seeded on top of these materials will attach and proliferate normally (Schaffer et al., 1994). Furthermore, these silicone membranes can foster both viability and proliferation of seeded ASCs (Razavi and Thakor, 2018). The ability to treat and coat silicone and other biomaterials remains an attractive method to promote differentiation and growth, and these parameters may be tuned to optimize proper cell growth.

The choice of polymer influences cell growth and differentiation through both growth factors and the stiffness of the matrix (Gilpin and Yang, 2017; Kargozar et al., 2020). For example, Poly(lactic-co-glycolic acid) (PLGA) has been extensively studied as one of the most widely used polymers for materials science engineering applications (Uematsu et al., 2005), including for cartilage and bone regeneration. Sadeghi-Avalshahr et al. (2017) seeded fibroblasts and keratinocytes in a scaffold made of a PLGA-collagen mix for dermal tissue engineering using electrospinning, a complicated process that produces physiologic ECM matrix structure with a low yield strength. Yang et al. (2018) recently created a biodegradable, inorganic MnO_2_ 3D nanoscaffold with a tunable, wide range of biodegradation times, upregulated ECM-protein binding affinities, highly efficient drug loading, and tunable drug release schedules. While they showed that their scaffold enhanced stem cell transplantation, differentiation, and drug delivery, their 3D nanoscaffolds also required a complex methodology to synthesize.

### Advantages of Hydrogel Scaffolds to Deliver Cell Therapies

While scaffolds developed with synthetic polymers or from decellularization can produce ECM constructs with high tunability and physiologic tissue structure, they require difficult methodology and testing to procure and develop, with elevated cost, excessive cellular adhesion, and slow scaffold degradation rates. Many of those scaffolds were designed to completely replace a damaged organ, meaning that any seeded cells within the constructs remain there over long time periods instead of leaving the construct to enter a wound. Using these scaffolds for wound care would be especially disadvantageous due to the need to clean and debride the wounds every several days.

**FIGURE 4:**
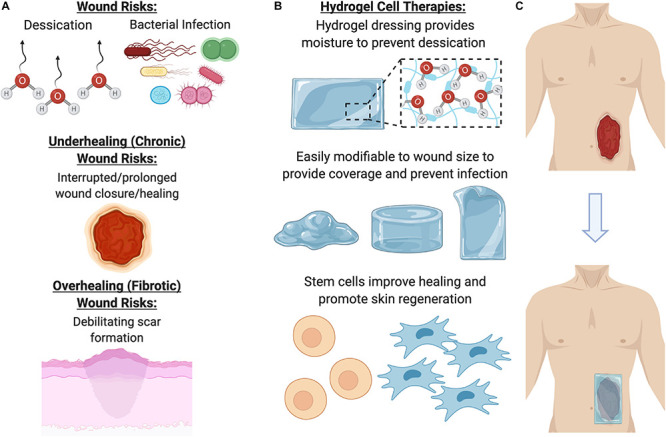
Benefits of using a hydrogel dressing to deliver cellular therapies. **(A)** Open wounds are at risk for desiccation (loss of moisture) and bacterial infection, as well either underhealing or overhealing. **(B)** To address these major risk factors, hydrogel dressings provide coverage and moisture, as well as a beneficial ECM environment for cells to grow in. **(C)** Once the hydrogel has been seeded with cells, it can be laid on the wound to promote healing and regeneration.

For wound care specifically, an ideal therapy would address concerns such as desiccation (loss of moisture from the wound), long term storage, bacterial infection, preventing debilitating scar formation, and promoting proper skin regeneration (growth of skin appendages, such as hair follicles, and other cutaneous glands) within the wound (Figure 4). Over the past decade, there has been increasing evidence that therapeutic hydrogels may address many of these concerns and promote natural skin regeneration, based on strong and promising laboratory and preclinical research findings. These dressings can be kept lyophilized (dry), making them lightweight, portable, and shelf stable. In the clinic, they can be simply unpackaged and soaked in saline to re-hydrate it, providing coverage across and preventing desiccation of the wound to facilitate healing.

Hydrogels have a unique set of properties which make them an ideal candidate for wound dressings. Their high water content confers physical similarity and biocompatibility to body tissues and maintains a moist environment around the wound interface (Kamoun et al., 2017; Youngblood et al., 2018). Hydrogel elasticity, mechanical properties, non-adhesion properties, and structural similarity to natural tissue also improve biocompatibility after implantation (da Silva et al., 2019). Hydrogels can be used as a supportive scaffold to deliver therapeutic cells safely to the wound site and shield the delivered cells from immune system attack while retaining permeability to therapeutic, signaling, and metabolic factors (Nafea et al., 2011). The hydrogel microenvironment can be tightly modified to support cells by adjusting numerous biophysical and biochemical properties, such as hydrogel–cell interactions, cell adhesion, microstructure, and degradability (Xu et al., 2020). Common hydrogel sources for wound healing include natural polymers like collagen, alginates, gelatin, and hyaluronic acid, as well as synthetic compounds such as polyethylene glycol and polyurethane (Liu et al., 2017).

### Current Clinical Use of Hydrogel Scaffolds and Cellular Therapies

Hydrogels can be commercially obtained as a sheet, gel, or saturated gauze, and some hydrogel products are already being used for wound care from companies such as Medline, McKesson, 3M, ConvaTec, Derma Sciences, or Smith & Nephew. These hydrogels are easy to use and apply to the wound surface. A secondary dressing is required over these hydrogels to secure them in place, which may be useful to prevent infection. For an infected, dry wound, physicians may also use hydrogels with antimicrobial silver incorporated within them, such as Silvasorb from Medline (Das et al., 2015). While these hydrogels are beneficial for most types of wounds, they are rarely used on already moist wounds, such as venous leg ulcers, as they may cause a high amount of output drainage and exudate from the site that further impedes healing by slowing down cell growth or degrading the tissue matrix structure (Murakami et al., 2010). Excess output draining may also promote inflammation or bacterial contamination. Overapplication of these hydrogels may also macerate or soften the skin surrounding the wound and reduce their integrity. Usually, these hydrogels are changed every 3 days, so production of these scaffolds must be simple, quick, and inexpensive to be commercially appealing for physicians.

While hydrogels have become commonplace within the clinic, the use of these hydrogels to house and deliver cells has not been FDA approved. It seems likely that combining the most efficient cell type—stem cells—with the most efficient bioactive scaffold for wound healing—hydrogels—would result in the most effective and beneficial therapy for wound care (da Silva et al., 2019).

### Designing Hydrogels for Cell Delivery

There are several technical considerations when designing a hydrogel for therapeutic cell delivery in wound healing. The most common method to incorporate cells within ECM scaffolds is through cellular encapsulation, in which cells are first suspended within a liquid precursor solution. Prior to hydrogel encapsulation, this liquid solution is buffered to the appropriate osmolarity to prevent cell lysis (Caliari and Burdick, 2016). The encapsulation process must be mild to not adversely impact cell viability. Hydrogels may also be pre-formed without cells, and then cells may be seeded on top of the hydrogels. The chemistry and structure of the hydrogel for both methods should facilitate cell proliferation and/or differentiation (Nicodemus and Bryant, 2008), and facilitating migration is especially important to promote seeded cells to crawl into the hydrogel matrix structure.

**FIGURE 5:**
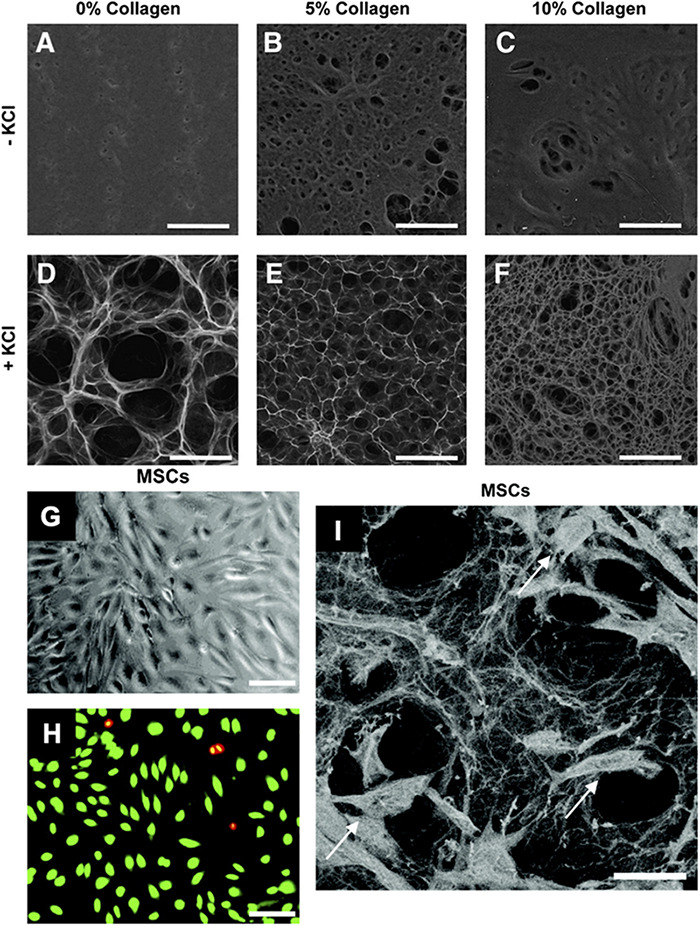
Pullulan-collagen hydrogel can be easily modified across a wide range of factors and provides biocompatibility with stem cells. **(A–F)** Scanning electron microscopy (SEM) imaging demonstrates that varying collagen and KCl concentrations significantly alters the porosity of the hydrogels. Hydrogels fabricated with KCl showed increased porosity, while increasing collagen concentrations decreased porosity. Scale bar 100 μm. **(G,H)** Brightfield imaging and fluorescent imaging of live (green) dead (red/yellow) stain shows successful *in vitro* cellular incorporation of MSCs in the hydrogels. Scale bar 50 μm. **(I)** SEM shows MSCs (arrows) viably incorporated within the pullulan-collagen hydrogels. Scale bar 25 μm. Figures adapted with permission from Figures 2, 6 of Wong et al. (2011b).

Hydrogels formed via gelation mechanisms require crosslinking of polymer chains by covalent, ionic, or physical bonds (Caliari and Burdick, 2016). Cells may become entrapped within hydrogels due to their micrometer size, as the three-dimensional structure of hydrogels contains a mesh size usually smaller than the size of the cell (nanometer scale) (Bae et al., 2014; Li and Mooney, 2016). Increasing hydrogel porosity will allow for more space to increase diffusion of nutrients, growth factors, trophic factors, and secreted ECM components from the surrounding medium to other cells throughout the matrix (Seliktar, 2012). More dense mesh size (nanoscale) will increase the concentration of cell–matrix interactions, which in turn promotes focal adhesion contacts and increased cellular adhesion. However, more porous structures (micro-scale) will facilitate migration throughout or even out of the construct into the wound area (Figures 5A–F; Wong et al., 2011b). Porosity of the ECM can be modulated with methods such as sacrificial beads, particle annealing, or addition or subtraction of potassium chloride (KCl) salt. Hwang et al. pre-formed scarified gelatin beads and mixed them with alginate to create hydrogels. The gelatin beads dissolved at physiologic temperatures, resulting in a porous alginate hydrogel (Hwang et al., 2010). Alison et al. (2019) also utilized these methods by coating corn oil droplets with silica nanoparticles and mixing these droplets together. After a drying process, the corn oil dissolved, leaving either a porous silica structure, and porosity could be modulated by modifying the size of the initial corn oil droplets (Alison et al., 2019). Griffin et al. (2015) created poly(ethylene) glycol–vinyl sulfone (PEG–VS) spherical particles and annealed them together to create microporous hydrogel materials. 3D printing may also be used to help create porous structures (Alison et al., 2019).

There are numerous factors which influence hydrogel degradation around encapsulated cells, including the cell type, hydrogel chemistry, and number of degradable linkages (Wong et al., 2011b, c; Rustad et al., 2012; Kosaraju et al., 2016). Biodegradable hydrogels formed from physical or ionic crosslinks are often degraded by a combination of hydrolysis or enzyme-mediated processes. In many cases, the degradation profile and rate can be controlled by adjusting parameters of the crosslinked structure. For instance, Wong et al. (2011b) cross-linked pullulan-collagen matrices with sodium trimetaphosphate (STMP) to increase strength and decrease degradation rate and found that the ratio of pullulan to collagen ECM affected the strength of STMP cross-linking. Henderson et al. (2010) and others have used ionic cross-linking, submerging their poly(methyl methacrylate) (PMMA) hydrogels into solutions with Zinc, Calcium, Nickel, Cobalt, and Copper. If hydrogel degradation occurs too quickly, the hydrogel-cell construct will dissolve before therapeutic benefit has been derived. If degradation occurs too slowly, a buildup of secreted factors may accumulate around encapsulated cells and negatively influence their function. In general, highly crosslinked gels possess longer degradation times. Cell mediated enzymatic degradation of hydrogels often occurs in hydrogels synthesized from natural biopolymers (e.g., hyaluronic acid-based hydrogels primarily degrade from cell-secreted enzymes like hyaluronidase). Short amino acid sequences, which are susceptible to enzymatic cleavage, may also be incorporated within the crosslinking of gels to accelerate degradation (Kong et al., 2004).

Ultimately, there are a multitude of technical considerations to account for when designing the optimal hydrogel for cell delivery. In order to successfully transition this therapy into the clinic, the process must be easily scaled for manufacturing and accepted by surgeons, patients, and healthcare providers. Cells pre-encapsulated within hydrogels may be hard to maintain viability, and these cells may also secrete factors that slowly degrade the hydrogel material properties. Seeding premade hydrogels with cells is especially clinically translatable, since cells could be cultured separately and then added to the hydrogel at the point of care. In addition, the un-seeded hydrogel must be easy to handle and encourage rapid cell seeding (Garg et al., 2014). Accounting for these technical considerations is crucial to facilitate the successful transition of hydrogel-cell therapies from laboratory research through FDA approval and into the clinical setting (Figure 4).

### Types of Hydrogels to Deliver Cells for Wound Healing

The base materials used for hydrogel construction for wound healing applications can generally be divided into two categories: natural polymers and synthetic polymers. The advantage of synthetic polymers such as poly-(ethylene glycol) [PEG] lie in their versatility for chemical modification and subsequent ability to finely tune the mechanical properties (Zhu and Marchant, 2011). However, since synthetic hydrogels lack the biochemical properties for cellular interaction, they are often synthesized in combination with natural polymers or biomimetic peptides. Examples of biocompatible natural polymers include chitosan, hyaluronic acid, heparin, alginate, fibrin, and collagen. The mechanical and biochemical properties of these materials are able to facilitate key functions for tissue regeneration including cell adhesion and migration (Zhu and Marchant, 2011).

Hyaluronic acid (HA) is a biocompatible glycosaminoglycan found in the extracellular matrix of connective and generally synthesized through bacterial fermentation, although it may also be sourced from animal products such as rooster combs. HA-based biomaterials degrade *in vivo* in response to hyaluronidase and have been used for a multitude of biomedical applications, since they possess several attractive hydrogel properties due to chemical tunability (Baier Leach et al., 2003). HA can also be modified to present functional groups, enabling a variety of crosslinking chemistries to produce a variety of different hydrogel types, including two-dimensional films, injectable materials, and three-dimensional free-swelling hydrogels. Silva et al. (2016) recently utilized HA-based, spongy hydrogels seeded with hASCs for application in a diabetic mouse full-thickness wound model and their results showed accelerated wound closure and neoinnervation. This study and others have illustrated the favorable mechanical properties, biocompatibility, and biodegradation capacity of HA based hydrogels for application in wound healing.

Alginate is a cationic biopolymer obtained from brown algae that has been utilized for hydrogel synthesis and previously used in multiple biomedical applications, including in wound healing (Salehi et al., 2020; Zhang and Zhao, 2020). Alginate forms physically crosslinked hydrogels in the presence of divalent cations (Percival and McCarty, 2015; Aderibigbe and Buyana, 2018), and the mechanical properties of the resulting hydrogel can be tuned by varying the polymer count, molecular weight, and concentration of cations capable of crosslinking. To enable cell attachment, alginate must be modified with an adhesive ligand, in contrast to other natural hydrogels like collagen and fibrin which do not require modification to support cell adhesion (Aderibigbe and Buyana, 2018; Tavakoli and Klar, 2020). One important drawback of an alginate-based hydrogel is its limited long-term stability in physiologic conditions, as these hydrogels can be dissolved due to ion exchange reactions with monovalent cations in the environment (Lee and Mooney, 2012). Zhang et al. (2020) recently developed sodium/alginate hydrogels to encapsulate human umbilical cord derived MSCs (hUC-MSCs). Their results showed that their alginate-based hydrogel established a robust microenvironment for hUC-MSCs to exert their therapeutic effects *in vivo*.

Gelatin has been investigated as a potentially promising polymer backbone for hydrogel synthesis due to its biocompatibility, biodegradability, and ease of chemical modification (Kang and Park, 2021). This material is conventionally extracted from porcine, bovine, or fish collagen. In the context of wound healing, gelatin-based hydrogels have gained attention as a promising substrate to synthesize *in situ* forming hydrogels. Various crosslinking strategies have been developed to synthesize gelatin-based hydrogels that do not dissolve at body temperature. These include thermal gelation, EDC reactions, Schiff base reactions, and enzyme-mediated crosslinking, among others (Campiglio et al., 2019; Zhang et al., 2019). Eke et al. (2017) successfully encapsulated ASCs within a UV-crosslinked biodegradable gelatin/HA hydrogel to accelerate wound healing. They showed that over 90% of ASCs encapsulated within their gelatin hydrogels survived after 21 days. Limitations of gelatin-based hydrogels include weak mechanical properties, variation between synthesized batches, and accelerated biodegradation compared to other hydrogel types.

Fibrin is a natural polymer formed during wound coagulation that is formed via the cleavage of fibrinogen by the serine protease thrombin (Janmey et al., 2009; Murphy et al., 2017). Fibrin molecules interact primarily through a series of disulfide bonds, although Factor XIIIa provides additional crosslinking and is also activated by thrombin (Moreno-Arotzena et al., 2015). Fibrin’s natural role as a matrix involved in hemostasis and wound healing makes it a promising vehicle for cell delivery, and fibrin can trigger encapsulated cells to secrete extracellular matrix components and reparative growth factors important in wound healing. However, fibrin can be especially susceptible to protease-mediated degradation (Ahmed et al., 2007).

Poly-(ethylene glycol) [PEG] is one of the most commonly used synthetic polymers for hydrogel synthesis due to its biocompatibility and hydrophilicity. PEG provides a relatively inert hydrogel base for the introduction of chemical modifications to promote cell–cell interactions. The precursor PEG may be modified with a variety of functional groups, including thiols, amines, and acrylates, which adds high customization and versatility when creating PEG hydrogels. Multiple research groups have developed PEG-based hydrogels for cell delivery and tissue regeneration in a variety of biomedical applications. Dong et al. (2017) developed a (PEG)-gelatin hydrogel derived from multifunctional PEG-based hyperbranched polymer and a thiolated gelatin, which could encapsulate and support murine ASCs. A murine wound healing study showed that the hydrogel significantly improved cell retention, enhanced angiogenesis, and accelerated wound closure. Griffin et al. (2015) also created synthetic, microporous annealed particle (MAP) hydrogels made of PEG–VS and found that these hydrogels promoted wound closure faster than non-porous hydrogels made of the same material. A followup study utilizing these PEG MAP hydrogels demonstrated that hMSCs could be encapsulated and incorporated within the hydrogels, and that further modification of functional groups could improve proliferation and cell function (Xin et al., 2020). This study and others suggest that PEG–based hydrogels can regulate stem cell behaviors in 3D culture and deliver cells for wound healing.

Collagen is the primary organic constituent of native ECM, making it a highly promising material for hydrogel synthesis and cell encapsulation (Chattopadhyay and Raines, 2014). Collagen hydrogels are generally composed of primarily type I collagen; however, other constituents such as glycosaminoglycans as well as type II and III collagen may also be incorporated (Helary et al., 2012; Stoica et al., 2020). These hydrogels are highly biocompatible and cytocompatible, amenable to cell adhesion without modification (Chattopadhyay and Raines, 2014). Collagen hydrogels are formed by raising the temperature and pH of solubilized collagen to initiate fibril self-assembly. If solubilized collagen is mixed with a cellular suspension before self-assembly, this can initiate an easy method to facilitate cellular encapsulation (Chen et al., 2018).

### Pullulan Collagen Hydrogels

Our group has engineered novel pullulan-collagen hydrogels with tunable, soft biomechanical properties and biocompatibility for cell-based therapy encapsulation. To develop a soft, biocompatible hydrogel that recapitulates the three-dimensional organization of the native ECM, we combined pullulan, a linear homopolysaccharide produced by the fungus *Aureobasidium pullulans* with type I collagen. This material was crosslinked with sodium trimetaphosphate under alkaline conditions, and potassium chloride salt (KCl) was used as a porogen for in-gel crystallization (Wong et al., 2011b). These unique engineered hydrogels provide several key advantages over traditional materials. First, pullulan-collagen hydrogels best approximate the porous ultrastructure of native reticular ECM based on comparison of fiber length and crosslink distance using a network extraction analysis. Moreover, altering the concentration of the collagen:pullulan ratio enables engineering of the mechanical properties such as hydrogel stiffness and effective porosity with relative ease. Additionally, we have conducted both *in vitro* and *in vivo* tests to demonstrate the biocompatibility of pullulan-collagen hydrogels. In *in vitro* settings, these hydrogels are able to support the growth of multiple cell types including fibroblasts, endothelial cells, and mesenchymal stromal cells, with minimal cytotoxicity (Figures 5G–I; Wong et al., 2011a). Further, in a murine subcutaneous implantation model, this bioscaffold demonstrates retention of reticular architecture and cellular infiltration, indicating minimal rejection and favorable biomaterial-tissue integration. In both humanized excisional wound and murine burn models, we found improvements in early wound healing in excisional wounds treated with hydrogels compared to untreated wounds (Barrera et al., 2021). The hydrogel-treated wounds not only healed faster, but also displayed reduced long-term fibrosis as evidenced by a reduction in myofibroblast activation. Specifically, cells such as fibroblasts may migrate into the soft hydrogel environment during healing. The ECM has low stiffness and could provide structural cues to these cells to become less fibrotic and more regenerative (Wong et al., 2011a).

By using capillary forces, cells can also be optimally seeded into hydrogels at the point of care. First, a cellular suspension is placed on top of a hydrophobic surface (parafilm wax paper); a dry hydrogel is then placed on top of this suspension, resulting in active absorption of cells into the pores of the scaffold through capillary, hydrophobic, and entropic forces (Garg et al., 2014). We compared this seeding method against centrifugal seeding or direct injection of cells into hydrogels. Scanning electron microscopy (SEM) showed that capillary force seeding had the most optimal seeding time and efficiency, as well as long-term cell survival and stability of hydrogel structure. Engrafted ASCs and MSCs were found to have over 96% viability within the hydrogels, indicating a beneficial environment conducive to cell growth, and the data suggested that the hydrogel preserved ASCs in a quiescent state and created a functional niche for this stem cell population, with concomitant maintenance of full cellular differentiation capacity (Figures 5G–I). Garg et al. (2014) also showed that ASC-hydrogels could significantly improve healing in murine burns, increasing wound vascularity and pro-angiogenic cytokine expression and decreasing scar formation.

Adipose-derived stromal cells seeded in the pullulan-collagen hydrogels improved healing in a murine burn model (Barrera et al., 2021). Burn wounds on the dorsum of mice were treated with either ASC-seeded or unseeded hydrogels (controls). Wounds treated with ASC-hydrogels had significantly reduced wound closure time, reduced scarring, reconstructed collagen networks, more wound vascularity, upregulation of pro-angiogenic cytokines such as *Cxcl12* and *Vegfa*, and downregulation of the fibrotic marker *Timp1*. The ASC-hydrogel group did significantly better across multiple experiments suggesting their superiority and additive therapeutic benefit in wound healing.

This enhanced delivery mechanism was further validated using a highly potent subset of ASCs with enhanced regenerative potential (Rennert et al., 2016). This subpopulation was identified with high specificity using two surface markers, DPP4 and CD55, which also expressed increased levels of general stem cell markers (CD34 and CD73) and genes associated with embryonic stem cells (GGT1). The regenerative potential of the DPP4^+^/CD55^+^ ASCs was then tested in a diabetic murine excisional wound healing model (Rustad et al., 2012). Briefly, two 6 mm-thickness cutaneous wounds were excised on either side of the midline of the murine dorsum, with each wound stented with silicone rings sutured in place to prevent wound contraction. Following wounding, a total of 5 × 10^5^ cells in 200 ml of saline was placed on 4 mm hydrogel disks. The ASC-hydrogel treatment demonstrated enhanced time to closure, and improved dermal recovery as compared to control. These findings suggest that this subpopulation of ASCs may have increased regenerative and wound healing potential (Rocchi et al., 2010; Rugg-Gunn et al., 2012; Kannan et al., 2014).

We further validated the biomimetic pullulan-collagen hydrogel scaffold in its ability to deliver bone marrow-derived MSCs in a murine excisional wound healing model (Rustad et al., 2012). Rustad et al. (2012) demonstrated that wounds treated with MSC-hydrogels had increased secretion of angiogenic cytokines and expression of transcription factors associated with maintenance of pluripotency and self-renewal (*Oct4, Sox2, Klf4*) as compared to controls. Wounds treated with MSC-seeded hydrogels also showed significantly accelerated healing and a return of skin appendages. Wounds treated with MSC-seeded hydrogels demonstrated significantly enhanced angiogenesis, which was associated with increased levels of VEGF and other angiogenic cytokines within the wounds. These data suggest that biomimetic hydrogels provide a functional niche capable of augmenting MSC regenerative potential and enhancing wound healing, further supporting the beneficial, additive abilities of pullulan-collagen hydrogel scaffolds for wound repair and regeneration.

## Future Directions

**FIGURE 6:**
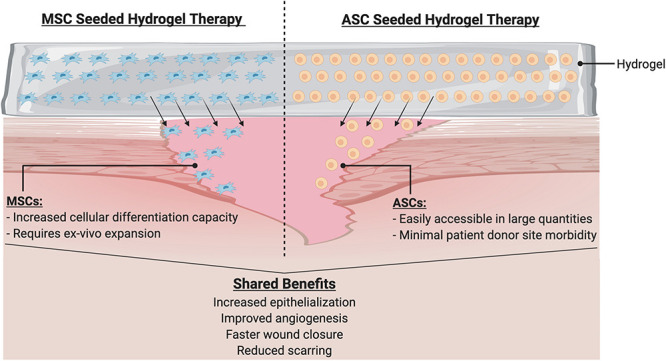
Hydrogel with cellular therapy promotes wound healing. Hydrogel delivery system serves as a physiologic milieu to encapsulate cells to ensure efficient delivery to wound site. Cells such as MSCs or ASCs may be seeded into the hydrogels. Upon application to the wound, cells migrate into the wound to initiate a cascade of beneficial phenotypes.

In this review, we have discussed the wide range of benefits associated with cell-based therapies for wound healing. There is substantial evidence that delivering cells to an injury site provides benefits and improvements to healing. Furthermore, the use of stem cells (both ASCs and MSCs) may be especially beneficial, due to these cells’ abilities to differentiate into the multitude of cell types needed in healthy tissue (Figure 6). Genetic engineering of these patient derived cells to enhance their therapeutic efficacy and instill novel cellular functions also represent a promising avenue for further investigation.

For tissue engineering, many researchers have explored methods to incorporate cells within extracellular matrices, with techniques ranging from materials science chemistry, decellularization, 3D printing, and electrospinning, to best create a physiologic ECM milieu for cell incorporation. While these techniques may be especially useful in cases where tissue must be replaced (heart, tendon, and liver), external wounds require therapies that provide both coverage and moisture to promote a proper healing environment, and soft hydrogels provide these baseline levels of beneficial healing. Cells seeded within these hydrogels adhere to the extracellular scaffold, but the adhesions are minimal enough so that cells may still detach and leave the hydrogel to enter the wound bed and encourage healing. This contrasts with other bioactive scaffolds that over promote adhesion so that cells remain within the scaffolds even at long time points.

Utilizing capillary forces, both ASCs and MSCs can be easily seeded within these hydrogels to improve viability and pro-regenerative phenotypes. This relatively straightforward seeding method can be readily translated for bedside application, as hydrogels and cells can be kept separately until just prior to application. It is likely that modifications to timing and frequency of the cellular treatment may improve these effects, as earlier or more frequent treatment may further reduce inflammation, fibrosis, and scarring, or further promote angiogenesis for healing. While a wide range of studies have explored cellular delivery or the use of hydrogels, additional work will be required to further optimize the parameters for these techniques. Further refinement and testing of these strategies in large animal models will also be helpful to eventually bring this technology to the bedside and facilitate clinical translation.

## Author Contributions

DS, KC, AC, DH, WW, CN, NM, SM, AM-B, CB, AT, JB, JP, MJ, and GG wrote the manuscript. All the authors contributed to the article andapproved the submitted version.

## Conflict of Interest

The authors declare that the research was conducted in the absence of any commercial or financial relationships that could be construed as a potential conflict of interest.

## References

[B1] Aderibigbe B. A. Buyana B. (2018). Alginate in wound dressings. *Pharmaceutics* 10:42. 10.3390/pharmaceutics10020042 29614804 PMC6027439

[B2] Adli M. (2018). The CRISPR tool kit for genome editing and beyond. *Nat. Commun.* 9:1911. 10.1038/s41467-018-04252-2 PMC5953931 29765029

[B3] Ahmed T. A. Griffith M. Hincke M. (2007). Characterization and inhibition of fibrin hydrogel-degrading enzymes during development of tissue engineering scaffolds. *Tissue Eng.* 13 1469–1477. 10.1089/ten.2006.0354 17518706

[B4] Alison L. Menasce S. Bouville F. Tervoort E. Mattich I. Ofner A. (2019). 3D printing of sacrificial templates into hierarchical porous materials. *Sci. Rep.* 9:409. 10.1038/s41598-018-36789-z PMC6344549 30674930

[B5] Atiyeh B. S. Gunn S. W. Hayek S. N. (2007). Military and civilian burn injuries during armed conflicts. *Annals Burns Fire Disasters* 20 203–215. PMC3188083 21991098

[B6] Bae H. Chu H. Edalat F. Cha J. M. Sant S. Kashyap A. (2014). Development of functional biomaterials with micro- and nanoscale technologies for tissue engineering and drug delivery applications. *J. Tissue Eng. Regen. Med.* 8 1–14. 10.1002/term.1494 22711442 PMC4199309

[B7] Baier Leach J. Bivens K. A. Patrick C. W. Jr. Schmidt C. E. (2003). Photocrosslinked hyaluronic acid hydrogels: natural, biodegradable tissue engineering scaffolds. *Biotechnol. Bioeng.* 82 578–589. 10.1002/bit.10605 12652481

[B8] Barrera J. Trotsyuk A. A. Maan Z. N. Bonham C. A. Larson M. R. Mittermiller P. A. (2021). Adipose-derived stromal cells seeded in pullulan-collagen hydrogels improve healing in murine burns. *Tissue Eng Part A.* Online ahead of print. 10.1089/ten.TEA.2020.0320 33789446

[B9] Berman B. Viera M. H. Amini S. Huo R. Jones I. S. (2008). Prevention and management of hypertrophic scars and keloids after burns in children. *J. Craniofacial Surg.* 19 989–1006. 10.1097/scs.0b013e318175f3a7 18650721

[B10] Bertozzi N. Simonacci F. Grieco M. P. Grignaffini E. Raposio E. (2017). The biological and clinical basis for the use of adipose-derived stem cells in the field of wound healing. *Ann. Med. Surg. (Lond).* 20 41–48. 10.1016/j.amsu.2017.06.058 28702186 PMC5491486

[B11] Block L. Gosain A. King T. W. (2015). Emerging therapies for scar prevention. *Adv. Wound Care (New Rochelle).* 4 607–614. 10.1089/wound.2015.0646 26487979 PMC4593896

[B12] Caliari S. R. Burdick J. A. (2016). A practical guide to hydrogels for cell culture. *Nat. Methods* 13 405–414. 10.1038/nmeth.3839 27123816 PMC5800304

[B13] Campiglio C. E. Contessi Negrini N. Fare S. Draghi L. (2019). Cross-Linking strategies for electrospun gelatin scaffolds. *Materials (Basel)* 12:2476. 10.3390/ma12152476 31382665 PMC6695673

[B14] Capelli C. Pedrini O. Valgardsdottir R. Da Roit F. Golay J. Introna M. (2015). Clinical grade expansion of MSCs. *Immunol. Lett.* 168 222–227. 10.1016/j.imlet.2015.06.006 26092523

[B15] Cargnoni A. Gibelli L. Tosini A. Signoroni P. B. Nassuato C. Arienti D. (2009). Transplantation of allogeneic and xenogeneic placenta-derived cells reduces bleomycin-induced lung fibrosis. *Cell Transplant.* 18 405–422. 10.3727/096368909788809857 19622228

[B16] Chattopadhyay S. Raines R. T. (2014). Review collagen-based biomaterials for wound healing. *Biopolymers* 101 821–833. 10.1002/bip.22486 24633807 PMC4203321

[B17] Chen J. M. Willers C. Xu J. Wang A. Zheng M. H. (2007). Autologous tenocyte therapy using porcine-derived bioscaffolds for massive rotator cuff defect in rabbits. *Tissue Eng.* 13 1479–1491. 10.1089/ten.2006.0266 17536925

[B18] Chen K. Vigliotti A. Bacca M. McMeeking R. M. Deshpande V. S. Holmes J. W. (2018). Role of boundary conditions in determining cell alignment in response to stretch. *Proc. Natl. Acad. Sci. U S A.* 115 986–991. 10.1073/pnas.1715059115 29343646 PMC5798351

[B19] Chen L. Tredget E. E. Liu C. Wu Y. (2009). Analysis of allogenicity of mesenchymal stem cells in engraftment and wound healing in mice. *PLoS One* 4:e7119. 10.1371/journal.pone.0007119 19771171 PMC2743192

[B20] Chidgey A. P. Layton D. Trounson A. Boyd R. L. (2008). Tolerance strategies for stem-cell-based therapies. *Nature* 453 330–337. 10.1038/nature07041 18480814

[B21] Chua A. W. Khoo Y. C. Tan B. K. Tan K. C. Foo C. L. Chong S. J. (2016). Skin tissue engineering advances in severe burns: review and therapeutic applications. *Burns Trauma* 4:3. 10.1186/s41038-016-0027-y PMC4963933 27574673

[B22] da Silva L. P. Reis R. L. Correlo V. M. Marques A. P. (2019). Hydrogel-Based strategies to advance therapies for chronic skin wounds. *Annu. Rev. Biomed. Eng.* 21 145–169. 10.1146/annurev-bioeng-060418-052422 30822099

[B23] Dabiri G. Damstetter E. Phillips T. (2016). Choosing a wound dressing based on common wound characteristics. *Adv. Wound Care (New Rochelle)* 5 32–41. 10.1089/wound.2014.0586 26858913 PMC4717498

[B24] Dabiri G. Heiner D. Falanga V. (2013). The emerging use of bone marrow-derived mesenchymal stem cells in the treatment of human chronic wounds. *Expert Opin. Emerg. Drugs* 18 405–419. 10.1517/14728214.2013.833184 24004161

[B25] Das A. Kumar A. Patil N. B. Viswanathan C. Ghosh D. (2015). Preparation and characterization of silver nanoparticle loaded amorphous hydrogel of carboxymethylcellulose for infected wounds. *Carbohydr Polym.* 130 254–261. 10.1016/j.carbpol.2015.03.082 26076624

[B26] Demidova-Rice T. N. Hamblin M. R. Herman I. M. (2012). Acute and impaired wound healing: pathophysiology and current methods for drug delivery, part 1: normal and chronic wounds: biology, causes, and approaches to care. *Adv. Skin Wound Care* 25 304–314. 10.1097/01.asw.0000416006.55218.d0 22713781 PMC3428147

[B27] Dong Y. Melanie Rodrigues S. A. Li X. Kwon S. H. Kosaric N. Khong S. (2017). Injectable and tunable gelatin hydrogels enhance stem cell retention and improve cutaneous wound healing. *Adv. Funct. Mater.* 27:1606619. 10.1002/adfm.201606619

[B28] Downing T. L. Soto J. Morez C. Houssin T. Fritz A. Yuan F. (2013). Biophysical regulation of epigenetic state and cell reprogramming. *Nat. Mater.* 12 1154–1162. 10.1038/nmat3777 24141451 PMC9675045

[B29] Edmonds M. (2009). Apligraf in the treatment of neuropathic diabetic foot ulcers. *Int. J. Low Extrem Wounds* 8 11–18. 10.1177/1534734609331597 19189997

[B30] Eke G. Mangir N. Hasirci N. MacNeil S. Hasirci V. (2017). Development of a UV crosslinked biodegradable hydrogel containing adipose derived stem cells to promote vascularization for skin wounds and tissue engineering. *Biomaterials* 129 188–198. 10.1016/j.biomaterials.2017.03.021 28343005

[B31] Falanga V. Iwamoto S. Chartier M. Yufit T. Butmarc J. Kouttab N. (2007). Autologous bone marrow-derived cultured mesenchymal stem cells delivered in a fibrin spray accelerate healing in murine and human cutaneous wounds. *Tissue Eng.* 13 1299–1312. 10.1089/ten.2006.0278 17518741

[B32] Farhat W. Hasan A. Lucia L. Becquart F. Ayoub A. Kobeissy F. (2019). Hydrogels for advanced stem cell therapies: a biomimetic materials approach for enhancing natural tissue function. *IEEE Rev. Biomed. Eng.* 12 333–351. 10.1109/rbme.2018.2824335 29993840

[B33] Field C. K. Kerstein M. D. (1994). Overview of wound healing in a moist environment. *Am. J. Surg.* 167:2S. 10.1016/0002-9610(94)90002-7 8109679

[B34] Fischbach M. A. Bluestone J. A. Lim W. A. (2013). Cell-based therapeutics: the next pillar of medicine. *Sci. Transl. Med.* 5:179s7. 10.1126/scitranslmed.3005568 PMC3772767 23552369

[B35] Frykberg R. G. Banks J. (2015). Challenges in the treatment of chronic wounds. *Adv. Wound Care (New Rochelle)* 4 560–582. 10.1089/wound.2015.0635 26339534 PMC4528992

[B36] Garg R. K. Rennert R. C. Duscher D. Sorkin M. Kosaraju R. Auerbach L. J. (2014). Capillary force seeding of hydrogels for adipose-derived stem cell delivery in wounds. *Stem Cells Transl. Med.* 3 1079–1089. 10.5966/sctm.2014-0007 25038246 PMC4149299

[B37] Geckil H. Xu F. Zhang X. Moon S. Demirci U. (2010). Engineering hydrogels as extracellular matrix mimics. *Nanomedicine (Lond)* 5 469–484. 10.2217/nnm.10.12 20394538 PMC2892416

[B38] Gentzkow G. D. Iwasaki S. D. Hershon K. S. Mengel M. Prendergast J. J. Ricotta J. J. (1996). Use of dermagraft, a cultured human dermis, to treat diabetic foot ulcers. *Diab. Care* 19:350. 10.2337/diacare.19.4.350 8729158

[B39] Gilpin A. Yang Y. (2017). Decellularization strategies for regenerative medicine: from processing techniques to applications. *Biomed. Res. Int.* 2017:9831534. 10.1155/2017/9831534 PMC5429943 28540307

[B40] Gonzalez A. C. Costa T. F. Andrade Z. A. Medrado A. R. (2016). Wound healing - a literature review. *An Bras Dermatol.* 91 614–620. 27828635 10.1590/abd1806-4841.20164741 PMC5087220

[B41] Griffin D. R. Weaver W. M. Scumpia P. O. Di Carlo D. Segura T. (2015). Accelerated wound healing by injectable microporous gel scaffolds assembled from annealed building blocks. *Nat. Mater.* 14 737–744. 10.1038/nmat4294 26030305 PMC4615579

[B42] Griffiths M. Ojeh N. Livingstone R. Price R. Navsaria H. (2004). Survival of apligraf in acute human wounds. *Tissue Eng.* 10 1180–1195. 10.1089/ten.2004.10.1180 15363174

[B43] Guo S. Dipietro L. A. (2010). Factors affecting wound healing. *J. Dent. Res.* 89 219–229. 20139336 10.1177/0022034509359125 PMC2903966

[B44] Gurtner G. C. Werner S. Barrandon Y. Longaker M. T. (2008). Wound repair and regeneration. *Nature* 453 314–321. 18480812 10.1038/nature07039

[B45] Hassan W. U. Greiser U. Wang W. (2014). Role of adipose-derived stem cells in wound healing. *Wound Repair Regen.* 22 313–325. 10.1111/wrr.12173 24844331

[B46] Helary C. Zarka M. Giraud-Guille M. M. (2012). Fibroblasts within concentrated collagen hydrogels favour chronic skin wound healing. *J. Tissue Eng. Regen. Med.* 6 225–237. 10.1002/term.420 22362469

[B47] Henderson K. J. Zhou T. C. Otim K. J. Shull K. R. (2010). Ionically cross-linked triblock copolymer hydrogels with high strength. *Macromolecules* 14 6193–6201. 10.1021/ma100963m

[B48] Hu M. S. Borrelli M. R. Lorenz H. P. Longaker M. T. Wan D. C. (2018). Mesenchymal stromal cells and cutaneous wound healing: a comprehensive review of the background, role, and therapeutic potential. *Stem Cells Int.* 2018:6901983. 10.1155/2018/6901983 PMC5985130 29887893

[B49] Hu M. S. Walmsley G. G. Barnes L. A. Weiskopf K. Rennert R. C. Duscher D. (2017). Delivery of monocyte lineage cells in a biomimetic scaffold enhances tissue repair. *JCI Insight.* 5:2. 10.1172/jci.insight.96260 PMC5841872 28978794

[B50] Hwang C. M. Sant S. Masaeli M. Kachouie N. N. Zamanian B. Lee S. H. (2010). Fabrication of three-dimensional porous cell-laden hydrogel for tissue engineering. *Biofabrication* 2:035003. 10.1088/1758-5082/2/3/035003 PMC3282162 20823504

[B51] Isakson M. de Blacam C. Whelan D. McArdle A. Clover A. J. (2015). Mesenchymal stem cells and cutaneous wound healing: current evidence and future potential. *Stem Cells Int.* 2015:831095. 10.1155/2015/831095 PMC4461792 26106431

[B52] Janmey P. A. Winer J. P. Weisel J. W. (2009). Fibrin gels and their clinical and bioengineering applications. *J. R. Soc. Interface* 6 1–10. 10.1098/rsif.2008.0327 18801715 PMC2575398

[B53] Jarbrink K. Ni G. Sonnergren H. Schmidtchen A. Pang C. Bajpai R. (2017). The humanistic and economic burden of chronic wounds: a protocol for a systematic review. *Syst. Rev.* 6:15. 10.1186/s13643-016-0400-8 PMC5259833 28118847

[B54] Kamoun E. A. Kenawy E. S. Chen X. (2017). A review on polymeric hydrogel membranes for wound dressing applications: PVA-based hydrogel dressings. *J. Adv. Res.* 8 217–233. 10.1016/j.jare.2017.01.005 28239493 PMC5315442

[B55] Kang J. I. Park K. M. (2021). Advances in gelatin-based hydrogels for wound management. *J. Mater. Chem. B* 9 1503–1520. 10.1039/d0tb02582h 33470270

[B56] Kannan N. Nguyen L. V. Eaves C. J. (2014). Integrin beta3 links therapy resistance and cancer stem cell properties. *Nat. Cell Biol.* 16 397–399. 10.1038/ncb2960 24914436

[B57] Kargozar S. Singh R. K. Kim H. W. Baino F. (2020). “Hard” ceramics for “Soft” tissue engineering: paradox or opportunity? *Acta Biomater.* 115 1–28. 10.1016/j.actbio.2020.08.014 32818612

[B58] Kim J. Y. Suh W. (2010). Stem cell therapy for dermal wound healing. *Int. J. Stem Cells* 3 29–31. 10.15283/ijsc.2010.3.1.29 24855538 PMC4022687

[B59] Kim W. S. Park B. S. Park S. H. Kim H. K. Sung J. H. (2009). Antiwrinkle effect of adipose-derived stem cell: activation of dermal fibroblast by secretory factors. *J. Dermatol. Sci.* 53 96–102. 10.1016/j.jdermsci.2008.08.007 18829265

[B60] Koenen P. Spanholtz T. A. Maegele M. Sturmer E. Brockamp T. Neugebauer E. (2015). Acute and chronic wound fluids inversely influence adipose-derived stem cell function: molecular insights into impaired wound healing. *Int. Wound J.* 12 10–16. 10.1111/iwj.12039 23490259 PMC7950656

[B61] Kong H. J. Alsberg E. Kaigler D. Lee K. Y. Mooney D. J. (2004). Controlling degradation of hydrogels via the size of cross-linked junctions. *Adv. Mater.* 16 1917–1921. 10.1002/adma.200400014 25067887 PMC4108267

[B62] Kosaraju R. Rennert R. C. Maan Z. N. Duscher D. Barrera J. Whittam A. J. (2016). Adipose-Derived stem cell-seeded hydrogels increase endogenous progenitor cell recruitment and neovascularization in wounds. *Tissue Eng. Part A* 22 295–305. 10.1089/ten.tea.2015.0277 26871860 PMC4779321

[B63] Kosaric N. Kiwanuka H. Gurtner G. C. (2019). Stem cell therapies for wound healing. *Expert Opin. Biol. Ther.* 19 575–585. 30900481 10.1080/14712598.2019.1596257

[B64] Lee K. Y. Mooney D. J. (2012). Alginate: properties and biomedical applications. *Prog. Polym Sci.* 37 106–126. 10.1016/j.progpolymsci.2011.06.003 22125349 PMC3223967

[B65] Li D. Wang A. Liu X. Meisgen F. Grunler J. Botusan I. R. (2015). MicroRNA-132 enhances transition from inflammation to proliferation during wound healing. *J. Clin. Invest.* 125 3008–3026. 10.1172/jci79052 26121747 PMC4563743

[B66] Li J. Mooney D. J. (2016). Designing hydrogels for controlled drug delivery. *Nat. Rev. Mater.* 1:16071. 10.1038/natrevmats.2016.71 PMC5898614 29657852

[B67] Lian X. Bao X. Al-Ahmad A. Liu J. Wu Y. Dong W. (2014). Efficient differentiation of human pluripotent stem cells to endothelial progenitors via small-molecule activation of WNT signaling. *Stem Cell Rep.* 3 804–816. 10.1016/j.stemcr.2014.09.005 25418725 PMC4235141

[B68] Liu W. Ma K. Kwon S. H. Garg R. Patta Y. R. Fujiwara T. (2017). The abnormal architecture of healed diabetic ulcers is the result of FAK degradation by calpain 1. *J. Invest. Dermatol.* 137 1155–1165. 10.1016/j.jid.2016.11.039 28082186

[B69] Ma K. Kwon S. H. Padmanabhan J. Duscher D. Trotsyuk A. A. Dong Y. (2018). Controlled delivery of a focal adhesion kinase inhibitor results in accelerated wound closure with decreased scar formation. *J. Invest. Dermatol.* 138 2452–2460. 10.1016/j.jid.2018.04.034 29775632

[B70] Meier K. Nanney L. B. (2006). Emerging new drugs for scar reduction. *Expert Opin. Emerg. Drugs* 11 39–47. 10.1517/14728214.11.1.39 16503825

[B71] Milan P. B. Lotfibakhshaiesh N. Joghataie M. T. Ai J. Pazouki A. Kaplan D. L. (2016). Accelerated wound healing in a diabetic rat model using decellularized dermal matrix and human umbilical cord perivascular cells. *Acta Biomater.* 45 234–246. 10.1016/j.actbio.2016.08.053 27591919 PMC5069185

[B72] Moon K. C. Suh H. S. Kim K. B. Han S. K. Young K. W. Lee J. W. (2019). Potential of allogeneic adipose-derived stem cell-hydrogel complex for treating diabetic foot ulcers. *Diabetes* 68 837–846. 30679183 10.2337/db18-0699

[B73] Moreno-Arotzena O. Meier J. G. Del Amo C. García-Aznar J. M. (2015). Characterization of fibrin and collagen gels for engineering wound healing models. *Materials (Basel)* 8 1636–1651. 10.3390/ma8041636 26290683 PMC4538789

[B74] Murakami K. Aoki H. Nakamura S. Nakamura S. Takikawa M. Hanzawa M. (2010). Hydrogel blends of chitin/chitosan, fucoidan and alginate as healing-impaired wound dressings. *Biomaterials* 31 83–90. 10.1016/j.biomaterials.2009.09.031 19775748

[B75] Murphy K. C. Whitehead J. Zhou D. Ho S. S. Leach J. K. (2017). Engineering fibrin hydrogels to promote the wound healing potential of mesenchymal stem cell spheroids. *Acta Biomater.* 64 176–186. 10.1016/j.actbio.2017.10.007 28987783 PMC5682213

[B76] Nafea E. H. Marson A. Poole-Warren L. A. Martens P. J. (2011). Immunoisolating semi-permeable membranes for cell encapsulation: focus on hydrogels. *J. Control Release* 154 110–122. 10.1016/j.jconrel.2011.04.022 21575662

[B77] Nagamura-Inoue T. He H. (2014). Umbilical cord-derived mesenchymal stem cells: their advantages and potential clinical utility. *World J. Stem Cells* 6 195–202. 10.4252/wjsc.v6.i2.195 24772246 PMC3999777

[B78] Nicodemus G. D. Bryant S. J. (2008). Cell encapsulation in biodegradable hydrogels for tissue engineering applications. *Tissue Eng. Part B Rev.* 14 149–165. 10.1089/ten.teb.2007.0332 18498217 PMC2962861

[B79] Nussbaum S. R. Carter M. J. Fife C. E. DaVanzo J. Haught R. Nusgart M. (2018). An Economic Evaluation of the Impact, Cost, and Medicare Policy Implications of Chronic Nonhealing Wounds. *Value Health* 21 27–32. 10.1016/j.jval.2017.07.007 29304937

[B80] Ogle M. E. Doron G. Levy M. J. Temenoff J. S. (2020). Hydrogel culture surface stiffness modulates mesenchymal stromal cell secretome and alters senescence. *Tissue Eng. Part A* 26 1259–1271. 10.1089/ten.tea.2020.0030 32628570

[B81] Ott H. C. Matthiesen T. S. Goh S. K. Black L. D. Kren S. M. Netoff T. I. (2008). Perfusion-decellularized matrix: using nature’s platform to engineer a bioartificial heart. *Nat. Med.* 14 213–221. 10.1038/nm1684 18193059

[B82] Percival S. L. McCarty S. M. (2015). Silver and alginates: role in wound healing and biofilm control. *Adv. Wound Care* 4 407–414. 10.1089/wound.2014.0541 26155383 PMC4486446

[B83] Pittenger M. F. Discher D. E. Peault B. M. Phinney D. G. Hare J. M. Caplan A. I. (2019). Mesenchymal stem cell perspective: cell biology to clinical progress. *NPJ Regen. Med.* 4:22. 10.1038/s41536-019-0083-6 PMC6889290 31815001

[B84] Raje N. Berdeja J. Lin Y. Siegel D. Jagannath S. Madduri D. (2019). Anti-BCMA CAR T-Cell therapy bb2121 in relapsed or refractory multiple myeloma. *N. Engl. J. Med.* 380 1726–1737. 31042825 10.1056/NEJMoa1817226 PMC8202968

[B85] Razavi M. Thakor A. S. (2018). An oxygen plasma treated poly(dimethylsiloxane) bioscaffold coated with polydopamine for stem cell therapy. *J. Mater. Sci. Mater. Med.* 29:54. 10.1007/s10856-018-6077-x PMC6190679 29725867

[B86] Reinke J. M. Sorg H. (2012). Wound repair and regeneration. *Eur. Surg. Res.* 49 35–43. 22797712 10.1159/000339613

[B87] Rennert R. C. Januszyk M. Sorkin M. Rodrigues M. Maan Z. N. Duscher D. (2016). Microfluidic single-cell transcriptional analysis rationally identifies novel surface marker profiles to enhance cell-based therapies. *Nat. Commun.* 7:11945. 10.1038/ncomms11945 PMC5512622 27324848

[B88] Rocchi A. Manara M. C. Sciandra M. Zambelli D. Nardi F. Nicoletti G. (2010). CD99 inhibits neural differentiation of human Ewing sarcoma cells and thereby contributes to oncogenesis. *J. Clin. Invest.* 120 668–680. 10.1172/jci36667 20197622 PMC2827943

[B89] Rose L. F. Chan R. K. (2016). The burn wound microenvironment. *Adv. Wound Care (New Rochelle)* 5 106–118. 10.1089/wound.2014.0536 26989577 PMC4779284

[B90] Rugg-Gunn P. J. Cox B. J. Lanner F. Sharma P. Ignatchenko V. McDonald A. C. (2012). Cell-surface proteomics identifies lineage-specific markers of embryo-derived stem cells. *Dev. Cell* 22 887–901. 10.1016/j.devcel.2012.01.005 22424930 PMC3405530

[B91] Rustad K. C. Gurtner G. C. (2012). Mesenchymal stem cells home to sites of injury and inflammation. *Adv. Wound Care (New Rochelle)* 1 147–152. 10.1089/wound.2011.0314 24527296 PMC3623614

[B92] Rustad K. C. Wong V. W. Sorkin M. Glotzbach J. P. Major M. R. Rajadas J. (2012). Enhancement of mesenchymal stem cell angiogenic capacity and stemness by a biomimetic hydrogel scaffold. *Biomaterials* 33 80–90. 10.1016/j.biomaterials.2011.09.041 21963148 PMC3997302

[B93] Sadeghi-Avalshahr A. Nokhasteh S. Molavi A. M. Khorsand-Ghayeni M. Mahdavi-Shahri M. (2017). Synthesis and characterization of collagen/PLGA biodegradable skin scaffold fibers. *Regen. Biomater.* 4 309–314. 10.1093/rb/rbx026 29026645 PMC5633691

[B94] Saghazadeh S. Rinoldi C. Schot M. Kashaf S. S. Sharifi F. Jalilian E. (2018). Drug delivery systems and materials for wound healing applications. *Adv. Drug Deliv. Rev.* 127 138–166. 10.1016/j.addr.2018.04.008 29626550 PMC6003879

[B95] Salehi M. Ehterami A. Farzamfar S. Vaez A. Ebrahimi-Barough S. (2020). Accelerating healing of excisional wound with alginate hydrogel containing naringenin in rat model. *Drug Deliv. Transl. Res.* 11 142–153. 10.1007/s13346-020-00731-6 32086788

[B96] Schaffer J. L. Rizen M. L’Italien G. J. Benbrahim A. Megerman J. Gerstenfeld L. C. (1994). Device for the application of a dynamic biaxially uniform and isotropic strain to a flexible cell culture membrane. *J. Orthop. Res.* 12 709–719. 10.1002/jor.1100120514 7931788

[B97] Schultz G. S. Chin G. A. Moldawer L. Diegelmann R. F. (2011). “Principles of wound healing,” in *Mechanisms of Vascular Disease: A Reference Book for Vascular Specialists*, eds Fitridge R. Thompson M. (Adelaide (AU): University of Adelaide Press).

[B98] Seliktar D. (2012). Designing cell-compatible hydrogels for biomedical applications. *Science* 336 1124–1128. 10.1126/science.1214804 22654050

[B99] Silva L. P. Pirraco R. P. Santos T. C. Novoa-Carballal R. Cerqueira M. T. Reis R. L. (2016). Neovascularization induced by the hyaluronic acid-based spongy-like hydrogels degradation products. *ACS Appl. Mater. Interfaces* 8 33464–33474. 10.1021/acsami.6b11684 27960396

[B100] Spalazzi J. P. Doty S. B. Moffat K. L. Levine W. N. Lu H. H. (2006). Development of controlled matrix heterogeneity on a triphasic scaffold for orthopedic interface tissue engineering. *Tissue Eng.* 12 3497–3508. 10.1089/ten.2006.12.3497 17518686

[B101] Srifa W. Kosaric N. Amorin A. Jadi O. Park Y. Mantri S. (2020). Cas9-AAV6-engineered human mesenchymal stromal cells improved cutaneous wound healing in diabetic mice. *Nat. Commun.* 11:2470. 10.1038/s41467-020-16065-3 PMC7235221 32424320

[B102] Stoica A. E. Chircov C. Grumezescu A. M. (2020). Hydrogel dressings for the treatment of burn wounds: an up-to-date overview. *Materials (Basel)* 25:13. 10.3390/ma13122853 PMC7345019 32630503

[B103] Strioga M. Viswanathan S. Darinskas A. Slaby O. Michalek J. (2012). Same or not the same? comparison of adipose tissue-derived versus bone marrow-derived mesenchymal stem and stromal cells. *Stem Cells Dev.* 21 2724–2752. 10.1089/scd.2011.0722 22468918

[B104] Sullivan D. C. Mirmalek-Sani S. H. Deegan D. B. Baptista P. M. Aboushwareb T. Atala A. (2012). Decellularization methods of porcine kidneys for whole organ engineering using a high-throughput system. *Biomaterials* 33 7756–7764. 10.1016/j.biomaterials.2012.07.023 22841923

[B105] Tartarini D. Mele E. (2015). Adult stem cell therapies for wound healing: biomaterials and computational models. *Front. Bioeng. Biotechnol.* 3:206. 10.3389/fbioe.2015.00206 26793702 PMC4707872

[B106] Tavakoli S. Klar A. S. (2020). Advanced hydrogels as wound dressings. *Biomolecules* 11:10. 10.3390/biom10081169 PMC7464761 32796593

[B107] Thamm O. C. Koenen P. Bader N. Schneider A. Wutzler S. Neugebauer E. A. (2015). Acute and chronic wound fluids influence keratinocyte function differently. *Int. Wound J.* 12 143–149. 10.1111/iwj.12069 23517467 PMC7950806

[B108] Thompson A. A. Walters M. C. Kwiatkowski J. Rasko J. E. J. Ribeil J. A. Hongeng S. (2018). Gene therapy in patients with transfusion-dependent beta-thalassemia. *N. Engl. J. Med.* 378 1479–1493. 29669226 10.1056/NEJMoa1705342

[B109] Trainor N. Pietak A. Smith T. (2014). Rethinking clinical delivery of adult stem cell therapies. *Nat. Biotechnol.* 32 729–735. 10.1038/nbt.2970 25093878

[B110] Uematsu K. Hattori K. Ishimoto Y. Yamauchi J. Habata T. Takakura Y. (2005). Cartilage regeneration using mesenchymal stem cells and a three-dimensional poly-lactic-glycolic acid (PLGA) scaffold. *Biomaterials* 26 4273–4279. 10.1016/j.biomaterials.2004.10.037 15683651

[B111] Williams D. F. (2009). On the nature of biomaterials. *Biomaterials* 30 5897–5909. 10.1016/j.biomaterials.2009.07.027 19651435

[B112] Wong V. W. Rustad K. C. Akaishi S. Sorkin M. Glotzbach J. P. Januszyk M. (2011a). Focal adhesion kinase links mechanical force to skin fibrosis via inflammatory signaling. *Nat. Med.* 18 148–152. 10.1038/nm.2574 22157678 PMC4457506

[B113] Wong V. W. Rustad K. C. Galvez M. G. Neofytou E. Glotzbach J. P. Januszyk M. (2011b). Engineered pullulan-collagen composite dermal hydrogels improve early cutaneous wound healing. *Tissue Eng. Part A* 17 631–644. 10.1089/ten.tea.2010.0298 20919949 PMC4398002

[B114] Wong V. W. Rustad K. C. Glotzbach J. P. Sorkin M. Inayathullah M. Major M. R. (2011c). Pullulan hydrogels improve mesenchymal stem cell delivery into high-oxidative-stress wounds. *Macromol. Biosci.* 11 1458–1466. 21994074 10.1002/mabi.201100180 PMC4157905

[B115] Wu Y. Chen L. Scott P. G. Tredget E. E. (2007). Mesenchymal stem cells enhance wound healing through differentiation and angiogenesis. *Stem Cells* 25 2648–2659. 10.1634/stemcells.2007-0226 17615264

[B116] Xiang J. Wang S. He Y. Xu L. Zhang S. Tang Z. (2019). Reasonable glycemic control would help wound healing during the treatment of diabetic foot ulcers. *Diab. Ther.* 10 95–105. 10.1007/s13300-018-0536-8 30465160 PMC6349287

[B117] Xin S. Gregory C. A. Alge D. L. (2020). Interplay between degradability and integrin signaling on mesenchymal stem cell function within poly(ethylene glycol) based microporous annealed particle hydrogels. *Acta Biomater.* 101 227–236. 10.1016/j.actbio.2019.11.009 31711899 PMC6960331

[B118] Xu X. Xia X. Zhang K. Rai A. Li Z. Zhao P. (2020). Bioadhesive hydrogels demonstrating pH-independent and ultrafast gelation promote gastric ulcer healing in pigs. *Sci. Transl. Med.* 26:12. 10.1126/scitranslmed.aba8014 32848095

[B119] Yang C. Tibbitt M. W. Basta L. Anseth K. S. (2014). Mechanical memory and dosing influence stem cell fate. *Nat. Mater.* 13 645–652. 24633344 10.1038/nmat3889 PMC4031270

[B120] Yang L. Chueng S. D. Li Y. Patel M. Rathnam C. Dey G. (2018). A biodegradable hybrid inorganic nanoscaffold for advanced stem cell therapy. *Nat. Commun.* 9:3147. 10.1038/s41467-018-05599-2 PMC6082841 30089775

[B121] Yao L. Bestwick C. S. Bestwick L. A. Maffulli N. Aspden R. M. (2006). Phenotypic drift in human tenocyte culture. *Tissue Eng.* 12 1843–1849. 16889514 10.1089/ten.2006.12.1843

[B122] You H. J. Han S. K. (2014). Cell therapy for wound healing. *J. Korean Med. Sci.* 29 311–319. 24616577 10.3346/jkms.2014.29.3.311 PMC3945123

[B123] Youngblood R. L. Truong N. F. Segura T. Shea L. D. (2018). It’s all in the delivery: designing hydrogels for cell and non-viral gene therapies. *Mol. Ther.* 26 2087–2106. 30107997 10.1016/j.ymthe.2018.07.022 PMC6127639

[B124] Zakrzewski W. Dobrzynski M. Szymonowicz M. Rybak Z. (2019). Stem cells: past, present, and future. *Stem Cell Res. Ther.* 10:68. 10.1186/s13287-019-1165-5 PMC6390367 30808416

[B125] Zhang L. Liu J. Zheng X. Zhang A. Zhang X. Tang K. (2019). Pullulan dialdehyde crosslinked gelatin hydrogels with high strength for biomedical applications. *Carbohydr. Polym.* 216 45–53. 31047081 10.1016/j.carbpol.2019.04.004

[B126] Zhang M. Zhao X. (2020). Alginate hydrogel dressings for advanced wound management. *Int. J. Biol. Macromol.* 162 1414–1428. 32777428 10.1016/j.ijbiomac.2020.07.311

[B127] Zhang Z. Li Z. Li Y. Wang Y. Yao M. Zhang K. (2020). Sodium alginate/collagen hydrogel loaded with human umbilical cord mesenchymal stem cells promotes wound healing and skin remodeling. *Cell Tissue Res.* 383 809–821. 33159581 10.1007/s00441-020-03321-7

[B128] Zhu J. Marchant R. E. (2011). Design properties of hydrogel tissue-engineering scaffolds. *Expert Rev. Med. Dev.* 8 607–626. 10.1586/erd.11.27 PMC3206299 22026626

[B129] Zhu Z. Ding J. Tredget E. E. (2016). The molecular basis of hypertrophic scars. *Burns Trauma.* 4:2. 10.1186/s41038-015-0026-4 PMC4963951 27574672

